# Perivascular Unit: This Must Be the Place. The Anatomical Crossroad Between the Immune, Vascular and Nervous System

**DOI:** 10.3389/fnana.2020.00017

**Published:** 2020-04-16

**Authors:** Fernanda Troili, Virginia Cipollini, Marco Moci, Emanuele Morena, Miklos Palotai, Virginia Rinaldi, Carmela Romano, Giovanni Ristori, Franco Giubilei, Marco Salvetti, Francesco Orzi, Charles R. G. Guttmann, Michele Cavallari

**Affiliations:** ^1^Department of Human Neuroscience, Sapienza University of Rome, Rome, Italy; ^2^Department of Medicine, Surgery and Dentistry, “Scuola Medica Salernitana”, Neuroscience Section, University of Salerno, Baronissi, Italy; ^3^Department of Neurology and Psychiatry, Sapienza University of Rome, Rome, Italy; ^4^Center for Neurological Imaging, Department of Radiology, Brigham and Women's Hospital, Harvard Medical School, Boston, MA, United States; ^5^Department of Neurosciences, Mental Health and Sensory Organs, Faculty of Medicine and Psychology, Centre for Experimental Neurological Therapies, Sapienza University, Rome, Italy; ^6^Department of Neurosciences Mental Health and Sensory Organs, Faculty of Medicine and Psychology, Sapienza University of Rome, Rome, Italy; ^7^IRCCS Istituto Neurologico Mediterraneo (INM) Neuromed, Pozzilli, Italy

**Keywords:** glymphatic system, perivascular space (PVS), neurodegenaration, neuroinflammation, amyloid, aquaporin (AQP)-4, blood brain barrier (BBB)

## Abstract

Most neurological disorders seemingly have heterogenous pathogenesis, with overlapping contribution of neuronal, immune and vascular mechanisms of brain injury. The perivascular space in the brain represents a crossroad where those mechanisms interact, as well as a key anatomical component of the recently discovered glymphatic pathway, which is considered to play a crucial role in the clearance of brain waste linked to neurodegenerative diseases. The pathological interplay between neuronal, immune and vascular factors can create an environment that promotes self-perpetration of mechanisms of brain injury across different neurological diseases, including those that are primarily thought of as neurodegenerative, neuroinflammatory or cerebrovascular. Changes of the perivascular space can be monitored in humans *in vivo* using magnetic resonance imaging (MRI). In the context of glymphatic clearance, MRI-visible enlarged perivascular spaces (EPVS) are considered to reflect glymphatic stasis secondary to the perivascular accumulation of brain debris, although they may also represent an adaptive mechanism of the glymphatic system to clear them. EPVS are also established correlates of dementia and cerebral small vessel disease (SVD) and are considered to reflect brain inflammatory activity. In this review, we describe the “perivascular unit” as a key anatomical and functional substrate for the interaction between neuronal, immune and vascular mechanisms of brain injury, which are shared across different neurological diseases. We will describe the main anatomical, physiological and pathological features of the perivascular unit, highlight potential substrates for the interplay between different noxae and summarize MRI studies of EPVS in cerebrovascular, neuroinflammatory and neurodegenerative disorders.

## The Perivascular Unit

### Overview

**Figure 1:**
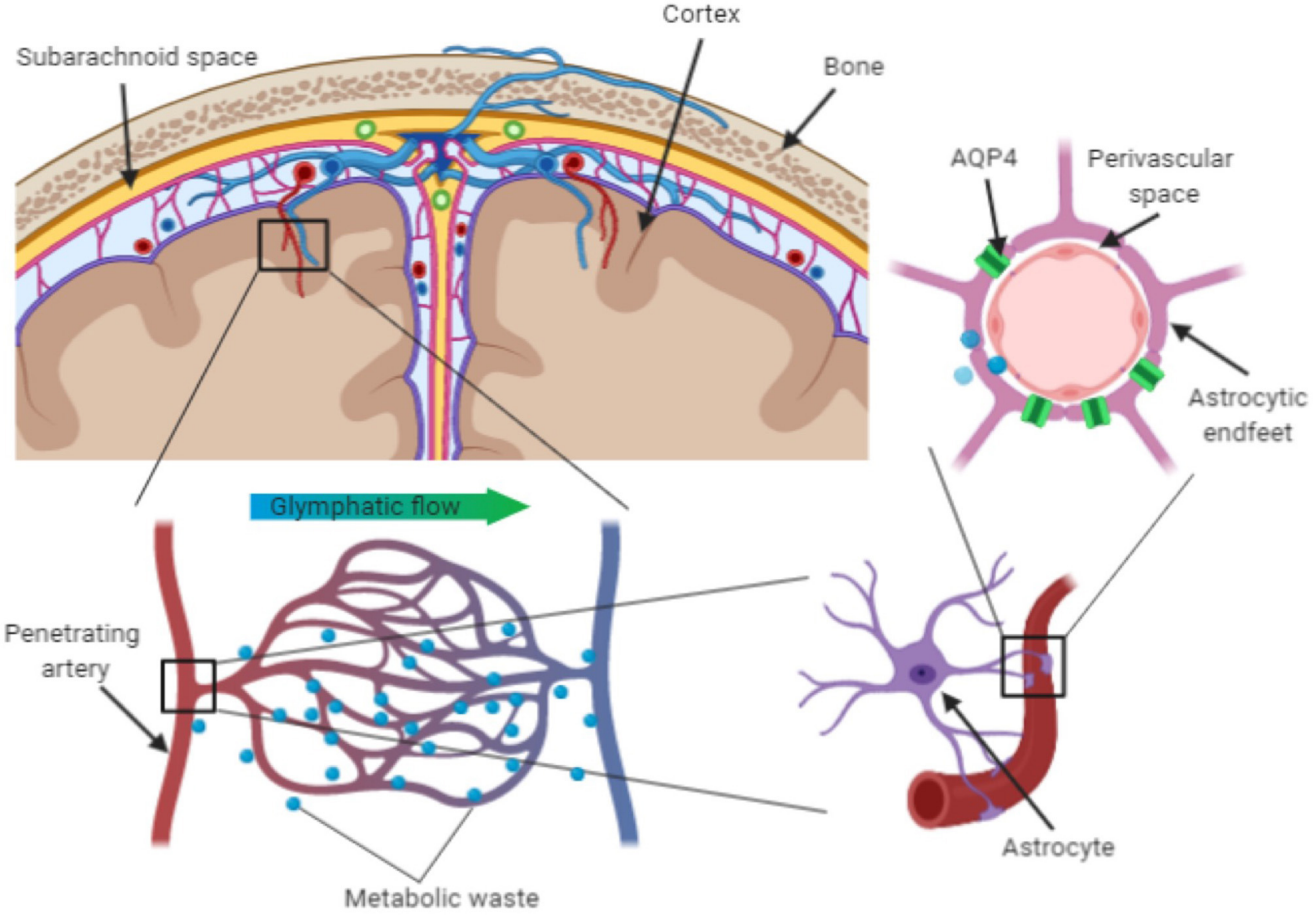
Schematic representation of perivascular unit.

We are adopting the term perivascular unit (PVU) in order to stress the contribution of cellular and molecular actors that surround the perforating vessels in the brain, and their interactions in determining the function the perivascular space (see Figure 1).

The perivascular space consists of a single or double layer of invaginated pia (Osborn and Preece, 2006), forming an interstitial fluid-filled space which represents the extension of the extracellular fluid space around the intracranial vessels as they dive into the brain parenchyma (Kwee and Kwee, 2007). The anatomy of perivascular spaces varies by location: in the centrum semiovale around superficial perforating arteries have only one layer of pia mater, while in the basal ganglia around the deep perforating arteries have two (Pollock et al., 1997).

Different factors have been associated with dynamic volume changes of the perivascular space, including vessel changes in diameter (vasoconstriction/vasodilation), neuronal activity, and neuronal and astrocytic swelling (Schain et al., 2017). A recent functional magnetic resonance imaging study (Fultz et al., 2019) suggests an alternation between blood and cerebrospinal fluid (CSF) flow during sleep, a condition known to be associated with glymphatic inflow of CSF through the perivascular space (Xie et al., 2013). Neural activity as measured by EEG preceded the CSF dynamics, thus suggesting that neural rhythms precede subsequent reduction in blood volume that allows CSF inflow through the perivascular space (Fultz et al., 2019).

### Cells of the PVU

The cellular components of the perivascular unit are mainly represented by neurons, but also include cells with predominant immune and support functions, such as endothelial cells, microglia, and pericytes.

As the pial and deep penetrating (lenticulostriate) arteries dive into the brain parenchyma, they gain pericytes between the endothelial cells and astrocyte endfeet. Along the length of the vessel, endothelial cells, neurons, pericytes, and astrocytes are in close proximity and their interaction constitutes the cellular substrate of the PVU function.

Endothelial cells lining the cerebral blood vessels represent one of the critical layers of the blood-brain barrier (BBB), which controls access of cells and molecules to the brain parenchyma. Transcytosis is limited by the lack of *fenestrae* in the endothelial cell layer (Iadecola, 2004) and paracellular transport of ions, macromolecules and other solutes is restrained by a tight interendothelial seal, mainly provided by tight junctions (Hawkins et al., 2006; Presta et al., 2018) made of transmembrane proteins coupled to the cytoskeletal actin (Stamatovic et al., 2016). As a result, most immunocompetent cells cannot access the brain parenchyma under physiological conditions, a phenomenon known as immune privilege.

The only exception is represented by the microglia, the resident brain macrophages that provide immuno-surveillance and first line defense against pathogens (Hickey and Kimura, 1988). During development, the microglia shape neural networks through synaptic pruning (Hong et al., 2016) and promote angiogenesis through interaction with endothelial progenitors (Engelhardt and Liebner, 2014). Microglial cells have small cell bodies and numerous long branching processes to survey the surrounding microenvironment for immune surveillance. Microglial cells are abundant in the surroundings of blood vessels and can be activated by pathogens and paracrine soluble factors released by activated monocytes and lymphocytes, as well as by the activated microglia themselves. When activated, the microglial cells change their morphology to larger nuclei and shorter processes, secrete numerous cytokines and soluble factors, and become highly phagocytic (Dudvarski Stankovic et al., 2016). Although the activated state of microglia is often referred to as a polarized phenotype—either pro- or anti-inflammatory—the spectrum of microglial states in response to pathogens and inflammatory stimuli appears to be much diverse than this dichotomous model would suggest (Mosser and Edwards, 2008). Activated microglia also release cytokines and chemokines that may result in increased BBB permeability and allow the migration of immune cells from the periphery into the central nervous system (CNS) (da Fonseca et al., 2014; Goldmann T. et al., 2016). Although the role of the microglia in neurological diseases is not completely understood, it can be summarized in two phenomena: one beneficial, with microglia acting as housekeeping phagocytes to maintain tissue homeostasis; another one harmful, with the microglia determining a pro-inflammatory state that results in synaptic dysfunction and increased secretion of potentially neurotoxic cytokines. Interestingly, both clinical and animal model studies revealed that microglial activation may occur at early stages of neurodegenerative, cerebrovascular, and neuroinflammatory diseases, such as Alzheimer's disease (AD), vascular dementia, and multiple sclerosis (MS) (Matsumoto et al., 1992; Wakita et al., 1994; Ihara et al., 2001; Ponomarev et al., 2005).

The resident microglia are part of the larger neuroglial cellular system, which includes non-neuronal cells of the nervous system, including astrocytes, oligodendrocytes, and pericytes. Those cells not only provide structural support to the brain parenchyma, but also respond to injury, regulate the ionic and chemical composition of the extracellular milieu, form the myelin insulation of the brain wiring, guide neuronal migration during development, and exchange metabolites with neurons (Verkhratsky and Steinhäuser, 2000).

Astrocytes constitute the physical bounding, with their end-foot processes, of the capillaries in the perivascular space. They extend their end-feet to the surface of cerebral blood vessels, providing 99% abluminal vessel coverage (Sosunov et al., 2014; Filosa et al., 2016). This position allows them to regulate cerebral blood flow in dynamic response to synaptic activity and neuronal metabolism (McConnell et al., 2017). Astrocytes can release vasoactive substances in response to neural activation, which adjust regional cerebral blood flow to provide an adequate supply of oxygen and nutrients to maintain the brain structural and functional integrity (Muoio et al., 2014). Astrocytes are also implicated in the glymphatic clearance of interstitial solutes linked to neurodegenerative processes, such as beta-amyloid and tau proteins. The trans-membrane water channel aquaporin-4 (AQP4) in particular plays a crucial role. Up to 50% of the brain aquaporin-4 (AQP4) is expressed on the astrocytes' endfeet and its density is higher in regions lining the cerebral ventricles (Jessen et al., 2015; Trevaskis et al., 2015; Plog and Nedergaard, 2018). Since AQP4 facilitates fluid exchanges between inflowing CSF and interstitial fluid, it is considered to play a key role in the clearance of potentially neurotoxic metabolites that accumulate in the interstitial space (Keep and Jones, 1990).

Experimental evidence supports that astrocytes promote myelination, as well as re-myelination after myelin injury (Domingues, 2016). During development, quiescent astrocytes may potentiate or prevent the differentiation of oligodendrocyte progenitor cells into mature myelinating oligodendrocytes. Astrocytes also contribute to the maintenance of myelin by stimulating myelin production in myelinating oligodendrocytes. In response to demyelinating injury, astrocytes secrete chemokines (e.g. CCL2 and CXCL10) to recruit activated microglia, which may further contribute to myelin injury (Moyon S Dubessy et al., 2015). Astrocytes and microglia can also promote remyelination after myelin injury, for instance by secreting chemokines (e.g. CXCL1, CXCL8 and CXCL10) to recruit oligodendrocyte progenitor cells to repopulate demyelinated regions (Omari et al., 2005).

Oligodendrocytes are best known for their role in forming the myelin sheets around neuronal axons that enable the saltatory conduction of neural signals across the cerebral white matter (Hamanaka et al., 2018). Oligodendrocytes also release growth factors for neighboring cells, as well as angiogenic factors associated with oligodendrogenesis of the adult white matter. Oligodendrocytes interact with other cells of the PVU, including pericytes, astrocytes and microglia. Their interaction with perycites is crucial for the development of the cerebral cortex and blood vessels (Choe et al., 2014; Uemura et al., 2018). The interaction between oligodendrocytes and microglia is crucial for myelination during brain development, as well as remyelination in demyelinating diseases, such as MS. One critical function of microglia in response to demyelination is the phagocytosis of myelin debris that would otherwise interfere with the recruitment and differentiation of oligodendrocyte progenitor cells (Kotter et al., 2006; Miron, 2017).

Pericytes are multifunctional mural cells wrapped around endothelial cells that line capillaries and venules. They are embedded in the basement membrane and communicate with endothelial cells by means of direct physical contact and paracrine signaling. Pericytes regulate capillary blood flow, the clearance and phagocytosis of cellular debris, and permeability of the blood–brain barrier (Wilhelm et al., 2016). Endothelial cells and pericytes are interdependent. The crosstalk between endothelial cells and pericytes is crucial for pericyte differentiation, recruitment and migration, but also for the maturation and stabilization of endothelial cells (Armulik et al., 2005). Communication failure between these two cell types is associated with several pathological conditions, including cardio- and cerebrovascular diseases, neurodegenerative diseases, and diabetic retinopathy. The platelet-derived growth factor signaling pathway of endothelial cells plays a key role in the crosstalk between pericytes and endothelial cells. Inhibition of this signaling pathway results in pericyte deficiency and endothelial hyperplasia, and is associated with diabetic retinopathy (Hammes et al., 2002).

### PVU and Glymphatic Clearance

The perivascular space has recently regained attention following the breakthrough discovery of the glymphatic pathway of clearance, of which the perivascular space is a crucial node (Xie et al., 2013). The glymphatic (glia + lymphatic) pathway of clearance has been named after the key role of glial cells and similarities with the lymphatic system (Nielsen et al., 1997; Nedergaard, 2013). Potentially neurotoxic soluble waste products are continuously released in the interstitial space due to the brain's high metabolic rate (Louveau et al., 2018) and the glymphatic pathway is considered a major route for the drainage of those metabolites.

Studies in animal models suggest that bulk flow of CSF enters the brain through the periarterial space and drains soluble waste products that accumulate in the interstitial space through the perivenous space to the subarachnoid space. From the subarachnoid space, those products are eliminated through the meningeal lymphatics to the extracranial lymphatic system (Tarasoff-Conway et al., 2015; Louveau et al., 2018). This mechanisms has been first described in rodents during sleep, when an observed 60% enlargement of the interstitial space associated with increased inflow of CSF through the perivascular space resulted in two times faster clearance of beta-amyloid (Xie et al., 2013), a protein implicated in the pathogenesis of AD. Although different mechanisms of beta-amyloid clearance have been demonstrated (including enzymatic degradation and cellular uptake, and transport across the blood-brain barrier), it has been estimated that 55–65% of the total extracellular beta-amyloid is eliminated through the glymphatic pathway (Tarasoff-Conway et al., 2015).

Hypothetically, a similar case may be made for soluble phase alpha-synuclein species in the brain but remains unproven at the present time. Given that patients with neurodegenerative disorders such as AD and Parkinson' disease (PD) have well-documented sleep disorders, it is not unreasonable to speculate that the sleep disturbances place an additional burden on an already dysfunctional protein folding and degradation system. Abnormal levels of alpha-synuclein are common to several neurodegenerative diseases (e.g., Lewy body disease, multiple system atrophy, PD), suggesting that aggregation of this protein is particularly neurotoxic and exacerbates neuronal degeneration (Yang et al., 2018; Parnetti et al., 2019). Though alpha-synuclein conformation change and accumulation were traditionally thought to occur in the neuron itself, recent evidence supports that alpha-synuclein is excreted into extracellular spaces (Emmanouilidou et al., 2011), making the neuropathological underpinnings of CSF-ISF flow in the pathogenesis of neurodegenerative disease processes is of high interest.

This led to some authors (Sundaram et al., 2019) proposing that glymphatic flow may be compromised due to the combined neurotoxic effects of alpha-synuclein protein aggregates and deteriorated dopaminergic neurons that are linked to altered REM sleep, circadian rhythms, and clock gene dysfunction (Gjerstad MD et al., 2008; Cai et al., 2010; Breen et al., 2014). Nevertheless, the observation that anesthesia increases glymphatic influx and efflux (Xie et al., 2013), suggests that it is not circadian rhythm but rather the sleep-wake state itself that determines the volume of the interstitial space and therefore the efficiency of glymphatic solute clearance. In this context, locus coeruleus–derived adrenergic signaling in the awake state seems to modify cell volume and thus the size of the interstitial space.

The glymphatic pathway has been best characterized in animal models. Studies investigating the glymphatic mechanism of clearance in humans are very limited. However, preliminary findings support the presence of a similar mechanism of clearance in humans. A small positron emission tomography study showed reduced beta-amyloid clearance through the cerebrospinal fluid in 8 AD patients compared to 7 healthy controls, as well as an association between brain deposition of beta-amyloid and reduced clearance (de Leon et al., 2017). An association between beta-amyloid deposition and reduced CSF turnover has been described in 20 subjects with normal pressure hydrocephalus (de Leon et al., 2017). Reduced beta-amyloid clearance through the CSF was also described in a small study of 8 AD patients (Ringstad et al., 2017). A functional MRI study showed large-amplitude pulsatile flow of CSF associated with non-REM sleep, possibly reflecting CSF dynamics associated with the glymphatic mechanisms of clearance described in animal models (Fultz et al., 2019).

## The PVU in Neurological Diseases

The etiology of most neurological diseases is unknown, and they are often described as multifactorial. The search for a *primum movens*—neuronal, vascular or immune-mediated—has been long considered critical to identify “the mechanism” to be targeted by early therapeutic interventions aimed at effectively contrasting the development and progression of the disease. However, remarkable overlapping of those mechanisms of brain injury at very early stages of disease pathogenesis has been demonstrated in many neurological diseases. A prototypical example regards the pathogenesis of AD, which was initially considered of vascular origin based on histopathological evidence of beta-amyloid deposits in the vessel wall (Sweeney, 2018). Over the course of the last century, different hypotheses of AD pathogenesis have emerged. Evidence of neurodegenerative processes associated with the progression of the disease contributed to ascribe the disease to a primarily neuronal etiology, as opposed to the initial vascular hypothesis. The etiology of AD is currently still debated and the overlapping contribution of vascular, neurodegenerative, and inflammatory mechanisms of brain injury from the very early stage has emerged as a key feature of AD pathogenesis (Iadecola and Gorelick, 2003). Another example is the emerging role of vascular factors in the formation of MS lesions—a disease that is considered primarily immune-mediated (Spencer et al., 2018).

The PVU represents a crossroad for the interaction of neurodegenerative, vascular, and immune mechanisms of brain injury. Here we describe those overlapping mechanisms, with particular focus on the cross-link between glymphatic, vascular and immune mechanisms potentially leading to neurodegenerative processes. A better understanding of the interaction between those mechanisms may lead to novel therapeutic approaches targeting combined factors, which may arguably be more effective in contrasting the development and progression of neurological diseases.

### Glymphatic Dysfunction

The glymphatic pathway has emerged as a key mechanism of clearance of Aβ and tau proteins (Iliff et al., 2012; Xie et al., 2013), the two hallmarks of AD pathobiology. According to the amyloid cascade hypothesis (Hardy and Higgins, 1992), the deposition of Aβ in the form of plaques is considered an early event in the pathogenesis of AD. Since a remarkable amount of Aβ is eliminated through the glymphatic pathway (Iliff et al., 2012, 2013; Xie et al., 2013), impaired glymphatic clearance could be implicated in the development and progression of AD. Interestingly, Aβ has detrimental effect on the glymphatic clearance, thus potentially leading to a deleterious feedback loop toward further impaired drainage and neurodegeneration (Carare et al., 2008; Iliff et al., 2012).

### Relationship Between Glymphatic and Vascular Abnormalities

Glymphatic dysfunction may be further impaired by vascular factors, including arteriosclerosis, vascular tortuosity, reduction in smooth muscle cell efficiency, and Aβ deposition in the vessel wall (Kalaria, 2002; Thore et al., 2007; Hawkes et al., 2011; Arbel-Ornath et al., 2013; Weller et al., 2015). Damage to the brain vasculature can lead to loss of perivascular integrity and AQP4 polarization (Iliff et al., 2012), as well as impairment of cerebrovascular pulsatility (Hadaczek et al., 2006), which is believed to facilitate CSF flow through the perivascular space. In older individuals, insoluble fibrillar Aβ deposits in the basement membranes of the walls of cerebral capillaries and arteries with the same spatial distribution of the perivascular pathways outlined by the injection of fluorescent tracers in the mouse brain (Weller et al., 2008). The initial deposition and subsequent accumulation of Aβ occurs in the lamina densa in the center of basement membranes surrounding smooth muscle cells in the artery wall. This may further disrupt perivascular drainage and thus exacerbate the impaired clearance of Aβ and other soluble waste from the aging brain (Hawkes et al., 2011, 2013).

### Vascular Abnormalities, Blood-Brain Barrier Permeability and Neuroinflammation

Pericytes and endothelial cells have a key structural role within the PVU. In animal models with lower pericyte coverage it has been observed an increased traffic of molecules across endothelial cells, which may enable proteins that would not have access to the brain in physiological conditions to enter the brain and potentially trigger an inflammatory response (Armulik et al., 2010). In a histopathological study of patients with AD, a significant loss of pericytes associated with BBB leakage was observed in the hippocampus and frontal cortex (Sengillo et al., 2013). In mice with AD-like pathology, loss of pericytes was observed in both the cortex and the hippocampus, while progressive perivascular dissociation of pericytes was characteristic to the hippocampus (Janota et al., 2015). Interestingly, pericytes have been described to migrate form the vessel wall during several pathological conditions (e.g., tumors, diabetic microangiopathy) (Ferland-McCollough et al., 2017). Although the pathogenic significance of pericyte dropout from the vessel wall is currently unknown, it has been hypothesized that this phenomenon is linked to increased endothelial and BBB permeability (Armulik et al., 2010).

In pathological conditions associated with increased BBB permeability, including neurodegenerative (e.g., AD), vascular (e.g., small vessel disease) and neuroinflammatory diseases (e.g., MS), T cells may enter the brain and encounter activated antigen presenting cells (Engelhardt et al., 2016), which are strategically localized in the perivascular space (Greter et al., 2005). Antigen presenting cells can induce proliferation and differentiation of encephalitogenic T cells, which release pro-inflammatory cytokines and trigger parenchymal invasion of other immune cells (Kawakami et al., 2004). This is well-established in EAE models where the recognition of the antigen expressed on antigen presenting cells is a fundamental prerequisite for the migration into the CNS of CD4+ T cells that trigger immune-mediated injury responsible for the clinical manifestations of the disease (Engelhardt et al., 2016).

### Relationship Between Glymphatic and Immune-Mediated Mechanisms of Brain Injury

The glymphatic clearance is tightly linked to the lymphatic system, in addition to the brain vasculature and perivascular space. The negative pressure that allows glymphatic flow is considered to derive from the meningeal lymphatics, which adsorb the subarachnoid CSF and drive soluble brain waste to the extra-cranial lymph nodes (Kipnis, 2016). The perivascular-lymphatic pathway represents the main route for drainage of antigens from the brain to cervical lymph nodes (Engelhardt and Coisne, 2011), with implications for immune tolerance and autoimmunity (Wolvers et al., 1999; Furtado et al., 2008; van Zwam et al., 2009). Specialized antigen presenting cells, such as macrophages, dendritic cells and T cells, are abundant in the leptomeninges and perivascular space, and they travel to the deep cervical lymph nodes through the same route of CSF flow, under both physiological and pathological conditions (Louveau et al., 2018). Several studies showed presence of T cells, antigen presenting cells and myelin and axonal antigens in cervical lymph nodes, after axonal injuries or autoimmune demyelination (de Vos et al., 2002; Fabriek et al., 2005; Goldmann J. et al., 2006; Hatterer et al., 2006; Engelhardt et al., 2016). In experimental autoimmune encephalomyelitis models (EAE, an animal model of MS) it has been shown that surgical or pharmacological blockage of lymphatic functions results in an attenuation of the disease (Louveau et al., 2018).

We have previously discussed how astrocytic AQP4 has a central role in the glymphatic mechanism of clearance of brain waste by facilitating water exchanges between the CSF and interstitial fluid. The role of AQP4 in neuroinflammatory conditions potentially linked to impaired glymphatic clearance has been investigated in EAE, as well as neuromyelitis optica (NMO), an inflammatory disease characterized by autoantibodies against AQP4 resulting primarily in complement-dependent astrocyte injury and secondarily in neuroinflammation, demyelination, and neuronal loss. Anti-AQP4 antibodies appear to alter the immunoregulatory function and barrier capacity of astrocytes (Hirt et al., 2018). In EAE, a loss of perivascular AQP4 localization has been reported (Wolburg-Buchholz et al., 2009). It is conceivable that impaired AQP4 function, due to either auto-antibodies (NMO) or mislocalization (EAE), may exert a deleterious effect on the glymphatic clearance, which ultimately contributes to the accumulation of neurotoxic solutes that may lead to neurodegeneration (Simon and Iliff, 2016; Plog and Nedergaard, 2018).

Furthermore, dopamine has been shown to reduce the proliferation of striatal glial cells and their expression of AQP4 (Küppers et al., 2008). Additionally, AQP-4 deficiency has been associated with enhanced susceptibility to insult of the dopaminergic neurons between the substantia nigra and ventral tegmental area (Zhang et al., 2016). Therefore, as dopaminergic neurons appear to modulate AQP4 function and AQP4 deficiency can exacerbate dopaminergic neuronal loss, the interplay of both processes may be impairing glymphatic system functioning and subsequently result in suboptimal clearance of alpha-synuclein in Parkinson's disease (PD).

The relationship between glymphatic dysfunction and inflammation has been studied in AD, a condition characterized by the brain deposition of Aβ in the form of plaques and tau proteins in the form of neurofibrillary tangles. Both Aβ and tau are eliminated through the glymphatic pathway, and their deposition in the brain is associated with synaptic dysfunction and neuronal loss. Aβ plaques and neurofibrillary tangles for their part can also trigger a secondary inflammatory response that may exacerbate neurodegeneration. Reactive astrogliosis and microglial activation surrounding amyloid plaques are established histopathological features of AD (Barger and Harmon, 1997). Aβ activates astrocytes and microglial cells through Toll-like receptor 4 (Reed-Geaghan et al., 2009). Activated astrocytes facilitate Aβ elimination across the BBB by low-density lipoprotein receptor-related protein 1 (Koistinaho et al., 2004), but excessive uptake of Aβ results in astrocyte dysfunction and apoptosis, leading to decreased clearance and increased accumulation of Aβ (Blanco et al., 2010). Interestingly, astrocyte activation is modulated by astrocytic AQP4, a protein that has a key role in the glymphatic mechanism of clearance. This further supports the link between glymphatic and immune-mediated mechanisms of brain injury and repair. Genetic ablation of AQP4 reduces the Aβ-induced activation of cultured astrocytes, leading to a reduction in the cellular uptake of Aβ and therefore contributing to worsen Aβ pathology (Yang et al., 2012). Activated microglia proliferate and overexpress genes coding for proinflammatory, potentially neurotoxic cytokines and receptors. Interestingly, studies in animal models showed that APO-E and TREM-2, two proteins implicated in determining the risk of cerebrovascular and neurodegenerative diseases, mediate the transition to neurodegeneration-like transcription profile in microglial cells (Krasemann et al., 2017). This further supports the overlap between neurodegenerative, cerebrovascular, and neuroinflammatory mechanisms of brain injury.

## Imaging the PVU in Humans

Although currently several efforts are underway to develop novel neuroimaging tools that may enable direct assessment of the glymphatic pathway of clearance (Ringstad et al., 2017; Eide et al., 2018), established methods to assess glymphatic flow in humans are not yet available. The perivascular space is a crucial node of the glymphatic pathway, and EPVS on MRI have recently emerged as biomarker of the pathway (Ramirez et al., 2016; Mestre et al., 2017). EPVS have been traditionally considered MRI correlates of small vessel disease (Wardlaw et al., 2013), have been shown to be associated with AD and small vessel disease in cross-sectional studies (Zhu et al., 2010; Wardlaw et al., 2013; Ramirez et al., 2015), and only recently have been proposed as MRI biomarker of the glymphatic pathway (Ramirez et al., 2016; Mestre et al., 2017). Based on the evidence of an association of EPVS with AD and small vessel disease, it has been speculated that EPVS may reflect glymphatic dysfunction or stasis. However, it remains unclear whether EPVS reflect glymphatic stasis or rather increased capacity of the perivascular space to drain potentially neurotoxic metabolites. To date, only one cross-sectional study examined the association between EPVS and brain deposition of beta-amyloid as measured by PET in 45 MCI and 69 sporadic AD subjects and found no association between EPVS and amyloid load (Banerjee et al., 2017). Longitudinal studies are warranted to clarify the temporal dynamics of EPVS in AD, and their relationship with biomarkers of amyloid and tau pathology over the course of the disease.

EPVS can be detected using conventional MRI sequences (such as T1 and T2-weighted pulse sequences) that are usually employed in clinical practice to diagnose and monitor neurological diseases. According to the STandards for Reporting Vascular Changes on NEuroimaging (STRIVE) criteria (Wardlaw et al., 2013), MRI-visible EPVS appear as fluid-filled spaces that follow the course of penetrating vessels as they enter the brain parenchyma. They appear as slit-like, round or ovoid structures, with diameter generally smaller than 3 mm, and signal intensity similar to the CSF. The assessment of EPVS on MRI mainly relies on visual methods to identify and count their number at specific brain locations where they are more likely to become apparent: the centrum semiovale, basal ganglia, hippocampus, and brainstem (Patankar et al., 2005; Adams et al., 2013, 2015; Potter et al., 2015). Automated methods for the detection and quantification of EPVS are being developed with the promise of improving sensitivity and robustness in detecting EPVS changes (Ballerini et al., 2018; Boespflug et al., 2018; Dubost et al., 2019).

### EPVS in Neurodegenerative and Cerebrovascular Diseases

Several studies have consistently shown associations of MRI-visible EPVS in the basal ganglia with hypertension, as well as with other correlates of cerebral small vessel disease, such as cerebral white matter hyperintensities, lacunes, microbleeds, and retinal vessel abnormalities (Rouhl et al., 2008; Doubal et al., 2010; Zhu et al., 2010; Klarenbeek et al., 2013; Wardlaw et al., 2013; Hansen et al., 2015; Mutlu et al., 2016; Ramirez et al., 2016; Arba et al., 2018). By contrast, MRI-visible EPVS in the cerebral white matter of the centrum semiovale are not typically associated with vascular risk factors, but are associated with advanced age (Doubal et al., 2010; Charidimou et al., 2013), AD (Roher et al., 2003; Chen et al., 2011; Banerjee et al., 2017) and cerebral amyloid angiopathy (Martinez-Ramirez et al., 2013; Charidimou et al., 2015, 2017; van Veluw et al., 2016).

The role of EPVS in the context of the glymphatic clearance of Aβ has been scrutinized in neuroimaging studies investigating the association between EPVS and Aβ deposition in cerebral amyloid angiopathy and AD. A recent study examined EPVS postmortem using MRI in five amyloid-positive brains and found a topographical association between EPVS and cortical amyloid deposition (van Veluw et al., 2016). Since no association was found between EPVS and amyloid plaques or vasculopathy related to cerebral amyloid angiopathy, the authors hypothesized that the deposition of Aβ in cortical and leptomeningeal vessels impairs the interstitial fluid clearance, resulting in retrograde enlargement of the perivascular space. Kasahara et al. (2019) found relatively higher EPVS in amyloid positron emission tomography (PET)-positive compared to PET-negative patients, although the differences were not significant. Banerjee et al. (2018) found that EPVS of the centrum semiovale were associated with amyloid PET positivity, although the association was not significant in analyses adjusted for demographics and vascular risk factors. Charidimou et al. (2014, 2015) reported that larger EPVS of the centrum semiovale were more frequent in patients with cerebral amyloid angiopathy compared to controls. In summary, to date the available literature does not provide compelling evidence of association between severity of EPVS and amyloid deposition as measured by PET (Table 1). One possible explanation for the lack of association between EPVS and amyloid PET findings might be the fact that, due to limited spatial resolution of PET imaging, PET-visible amyloid is considered to reflect parenchymal deposits, while MRI-visible EPVS may be considered a correlate of vascular dysfunction resulting from vascular amyloid deposits (Weller et al., 2015).

**Table 1:** Clinical and MRI correlates of perivascular spaces in previous structural neuroimaging studies on patients with Alzheimer's Disease.

	**Patankar et al. (2005)**	**Chen et al. (2011)**	**Martinez-Ramirez et al. (2013)**	**Hansen et al. (2015)**	**Ramirez et al. (2015)**	**Banerjee et al. (2017)**	**Ding et al. (2017)**	**Shams et al. (2017)**	**Banerjee et al. (2018)**
Number of subjects (Number of females)	AD: 35 (17) FTD: 16 (6) VaD: 24 (10) HC: 35 (24)	AD: 37 (23) MCI: 71 (26) HC: 50 (32)	AD: 37 CIND: 52	AD: 47 (N/A) VaD: 39 (N/A) HC: 65 (N/A)	AD: 203 (108) HC: 94 (53)	AD: 110 (61) VaD: 116 (67)	HC: 2612 (1542)*^##^*	AD: 423 (N/A) MCI: 418 (N/A) VaD: 54 (N/A) CAA: 98 (N/A) SCI: 385 (N/A)	AD: 69 (N/A) aMCI: 45 (N/A) VaD: 70 (N/A) svMCI: 67 (N/A)
Age (mean ± SD, year)	AD: 61.6 ± 7.01 FTD: 63.2 ± 9.24 VaD: 64.5 ± 7.35 HC: 72.8 ± 6.56	AD: 74.6 ± 9.0 MCI: 74.8 ± 7.6 HC: 75.5 ± 5.0	Mean age of participants: 72.7 ± 9.9^#^	AD: 74.1 ± 8.5 VaD: 76.9 ± 7.7 HC: 78 ± 5.6	AD: 72.7 ± 8.8 HC: 69.5 ± 7.8	AD: 70.3 ± 8.8 VaD: 73.8 ± 7.0	74.6 ±4.8	N/A^###^	Mean age of participants: 72.2 ± 8.1^####^
MRI scanner	1.5T	3T	3T	1.5T	1.5T	3T	1.5T	1.5T - 3T	3T
EPVS quantification	Manual EPVS count in frontal, parietal, and occipital periventricular regions; deep WM; BG and brainstem)	Manual EPVS scoring in 8 brain regions, including structures in the WM (subregions of the frontal lobe, temporal lobe, parietal lobe, occipital lobe, and the CS), BG, brainstem and hippocampal area	Established 4-point semiquantitative score in BG and WM	Semiquantitative EPVS scoring systems	Semiquantitative assessment of EPVS in BG and CS	Manual EPVS count in BG and CS	Visual rating	Visual rating in the CS and BG of EPVS	Visual rating in basal ganglia
Association of EPVS with AD	EPVS scores in both the BG and the CS were significantly higher in those with VaD than in patients with AD EPVS in the CS were significantly more frequent in patients with FTD than in HC.	Greater levels of EPVS in AD and MCI compared to HC	No apparent difference between the two groups	PVS dilation demonstrates greater specificity for discrimination among VaD, AD, and healthy individuals than white matter scoring schemes.	AD patients have significantly greater volumes of EPVS in the WM	Significantly higher CS EPVS count in clinically diagnosed AD and higher BG EPVS in clinically diagnosed VaD No association with PiB PET positivity.	Association with an increased risk of developing VaD but not with all-cause dementia or AD	BG-EPVS showed to be associated with high CSF tau in the whole cohort and in AD BG-EPVS was associated with VaD.	Presence of >10 BG-EPVS was included as an item of a SVD score, which was associated with deficits in frontal and visuospatial performance (across all participants)
Association of EPVS with clinical or demographic variables	N/A	Significant association with advanced age	Association of BG-EPVS with hypertension in patients with cognitive impairment	N/A	Greater volumes of total EPVS in men	N/A		CS- and BG-PVS were both associated with high age and hypertension	N/A
Association of EPVS with brain MRI variables	CS EPVS score positively correlated with atrophy in patients with FTD and VaD	Association of EPVS total score with leukoaraiosis and atrophy	Associations of WM-EPVS with lobar microbleeds in patients with cognitive impairment	N/A	N/A	N/A	Association of EPVS with increased risk of incident subcortical infarcts and microbleeds and greater WM hyperintensities progression	CS and BG-PVS were associated with probable CAA, moderate-to-severe WM hyperintensities, cortical superficial siderosis, cerebral microbleeds CS-PVS was separately associated with strictly lobar cerebral microbleeds. BG-PVS was associated with strictly deep cerebral microbleeds and lacunes.	Association of SVD score with reduced cortical thickness across a number of regions, in particular in frontal and superior temporal regions

# *In this study, the mean age of all participants and also the mean age of each subgroup according to the PVS count was expressed*.

## *This was a prospective, population-based cohort study that aimed to examine whether EPVS are associated with presence SVD neuroradiological markers and increased risk of dementia*.

### *In this study, mean age of all participants was expressed based on 4 subgroups according to the PVS count*.

#### *In this study, only the mean age of all participants was specified*.

*AD, Alzheimer's disease; aMCI, amnestic Mild Cognitive Impairment; svMCI, Subcortical vascular Mild Cognitive Impairment; BG, basal ganglia; CAA, Cerebral Amyloid Angiopathy; CIND, Cognitive Impairment With No Dementia; CS, Centrum Semiovale; EPVS, Enlarged Perivascular Spaces; FTD, Frontotemporal Dementia; HC, healthy control; MCI, Mild Cognitive Impairment; PiB PET, Pittsburgh compound B PET; SCI, Subjective Cognitive Impairment; VaD, Vascular Dementia; WM, White Matter*.

The link between EPVS and inflammation in neurodegenerative diseases has been investigated in recent studies reporting an association between EPVS and C-reactive protein in AD and small vessel disease (Satizabal et al., 2012; Hilal et al., 2018). Results from a study by Bi et al. (2012) suggest that C-reactive protein cytotoxicity is associated with Aβ formation. In rat adrenal pheochromocytoma cell lines, the levels of amyloid precursor protein, beta-site APP cleaving enzyme, and presenilins were found to be increased after treatment with subtoxic concentrations of C-reactive protein. Higher C-reactive protein levels were also associated with neuroimaging biomarkers of cerebral small vessel disease and higher plasma levels of Aβ1- 38, Aβ1-40, and Aβ1-42 (Hilal et al., 2018).

In the context of neurodegenerative disorders, parkinsonism with enlarged PVS in basal ganglia has been reported by several authors through the years (Fenelon et al., 1995; Mancardi et al., 1998; Duker and Espay, 2007; Mehta et al., 2013; Lee et al., 2015b). Their clinical significance remains unclear but in recent articles enlargement of PVS was found to be predictive of cognitive impairment in PD (Park et al., 2019; Shibata et al., 2019) and related to motor symptoms (Laitinen et al., 2000; Mestre et al., 2014; Lee et al., 2015a; Conforti et al., 2018; Wan et al., 2019). However, the validation of these observations is lacking, and the mechanisms by which enlarged PVS negatively affect motor and cognitive changes in PD remain largely unknown (see Table 2).

**Table 2:** Clinical and MRI correlates of perivascular spaces in previous structural neuroimaging studies on patients with Parkinson's Disease.

	**Laitinen et al. (2000)**	**Mestre et al. (2014)**	**Lee et al. (2015a)**	**Conforti et al. (2014)**	**Park et al. (2019)**	**Shibata et al. (2019)**	**Wan et al. (2019)**
Number of subjects (Number of females)	PD: 40	PD: 3 (1)	PD: 4 FTD: 1 PSP: 1 MSA: 2 VaP: 3	PD: 1 (1)	PD: 271 (N/A) [106 patients with intact cognition (PD-IC) and 165 patients with mild cognitive impairment (PD-MCI)]	PD: 71 (32)	PD: 137 (53)
Age (mean ± SD, year)	62.9	a 61-year-old left-handed man; a 41-year-old right-handed woman; a 52-year-old left-handed man	72	69	N/A	73.2 ± 8.4	68.1 ± 8.6
MRI scanner	1T	3T	N/A	3T	3T	1.5T	3T
EPVS quantification	Visual rating	Visual rating	Visual semi-quantitative scale (none=0, mild=1, moderate =2, severe=3) and a quantitative scale by counting the actual number of EPVS [0 (none), 1-9 =1 (mild), 10-19=2 (moderate), and ≥20=3 (severe)] in six segments (each striatum was divided into three segments: CN, AP and PP).	Semi- automated rating	PVS was rated in the BG and CS using a 4-point visual scale and then classified as high (score ≥ 2) or low (score <2) according to severity	Visual rating. Use of total SVD score (range: 0–4) based on white matter hyper intensities (WMHs), lacunae, cerebral microbleeds (MBs), and EPVS	Visual rating in BG and CS
Association of EPVS with PD	N/A	Single large EPVS contralateral to symptoms side	no significant correlation between the presence of EPVS and abnormality of the DaT-PET	Single giant EPVS in the left anterior perforated substance	In all patients, higher BG-PVS severity was an independent positive predictor of future cognitive decline. (mean follow-up of 5.0 ± 2.3 years)	No significant associations with total SVD score and PD severity and motor phenotype	N/A
Association of EPVS with clinical or demographic variables	Patients with predominantly right-sided symptoms had larger EPVS in the right compared with left *globus pallidus* and larger EPVS in the left putamen than in the right. The pattern for patients with predominantly left-sided symptoms was completely reversed. The size, site, and distribution of the EPVS did not correlate with the patients' age or sex, nor with duration of illness or HY stage	Hypothesized association of a single large EPVS may contribute to atypical clinical features in patients who otherwise had clinical/imaging findings consistent with idiopathic PD	The EPVS score was significantly correlated with K-MMSE, MoCA-K, and FAB, but not with HY stage	Symptoms affecting predominantly the right limbs	N/A	Increasing age and reduced MMSE and MoCA scores were associated with increased SVD burden	Association between EPVS in BG and the tremor score (p = 0.032)
Association of EPVS with brain MRI variables	N/A			N/A	N/A	Logistic regression analyses demonstrated that periventricular WM hyperintensities, PVS in BG and atrophy were predictors of cognitive impairment in PD	N/A

*AP, Anterior Putamen; BG, basal ganglia; CN, Caudate Nucleus; CS, Centrum Semiovale; EPVS, Enlarged Perivascular Spaces; FAB, Frontal Assessment Battery; FTD, Frontotemporal Dementia; HY, Hoehn and Yahr; K-MMSE, Korean—Mini Mental State Examination; MCI, Mild Cognitive Impairment; MoCA-K, Montreal Cognitive Assessment Korea; PD, Parkinson's disease; PP, Posterior Putamen; PSP, Progressive Supranuclear Palsy; SVD, Small Vessel Disease; VaP, Vascular Parkinsonism; WM, White Matter*.

### EPVS in MS

Although EPVS have been traditionally considered correlates of neurological diseases with primarily neurodegenerative or vascular pathogenesis, they have been also observed in neuroinflammatory diseases. Inflammatory cells accumulating in the perivascular space can damage the extracellular matrix, impair the integrity of BBB, and potentially lead to demyelination (Wong et al., 2013). Although the interaction between inflammatory cells, EPVS, and CSF dynamics has not yet been studied in detail, it is conceivable that the aggregation of inflammatory cells in the perivascular space could also obstacle the glymphatic clearance.

EPVS burden in MS was first assessed by Achiron and Faibel (2002) in a cohort of recent-onset MS patients. Most neuroimaging studies of EPVS in MS consistently showed that the number (Etemadifar et al., 2011; Conforti et al., 2014; Kilsdonk et al., 2015; Cavallari et al., 2018) and volume (Wuerfel et al., 2008) of EPVS was higher in MS patients compared to healthy controls (Table 3), with the exception of one study that failed to replicate these findings (Al-Saeed et al., 2012). The location of EPVS most consistently associated with MS was the centrum semiovale (Achiron and Faibel, 2002; Etemadifar et al., 2011; Kilsdonk et al., 2015; Cavallari et al., 2018). EPVS also appeared to be more frequent in atypical sites in MS patients compared to controls (Kwee and Kwee, 2007).

**Table 3:** Clinical and MRI correlates of perivascular spaces in previous structural neuroimaging studies on patients with multiple sclerosis.

	**Achiron and Faibel (2002)**	**Wuerfel et al. (2008)**	**Etemadifar et al. (2011)**	**Al-Saeed et al. (2012)**	**Conforti et al. (2014)**	**Kilsdonk et al. (2015)**	**Cavallari et al. (2018)**
Number of subjects (Number of females)	MS: 71 (47) HC: 60 (38)	MS: 45 (23) HC: 30 (16)	MS: 73 (55) HC: 73 (55)	MS: 80 (N/A) HC: 80 (N/A)	MS: 40 (28) HC: 30 (17)	MS: 34 (22) HC: 11 (6)	MS: 60 (48) HC: 15 (9)
Phenotype	N/A	45 RRMS	N/A	N/A	40 RRMS	22 RRMS 5 SPMS 7 PPMS	C MS: 30 (24) NC MS: 30 (24)
Age (mean ± SD, year)	MS: 26.8 ± 9.2 C: 27.2 ± 8.4	MS: 39.8 ± 8.2 C: 37.8 ± 11.5	MS: 32.3 ± 9.3 HC: 33.3 ± 10.0	MS: 15-49^#^ HC: N/A	MS: 42.7 ± 8.0 C: 42.8 ± 8.9	MS: 43.0 ± 7.9 C: 38.8 ± 10.5	C MS: 50 (27-68)^##^ NC MS: 48 (27-63)^##^ HC: 38 (24-56)*^##^*
Disability^*^ (mean ± SD)	N/A	2.3 ± 1.4	N/A	N/A	(1-6.5)^**^	4 (0-7.5)^##^	C MS: 2 (0-2) ^##^ NC MS: 0 (0-2)*^##^*
Disease duration (mean ± SD, year)	<0.3	8.8 ± 6.2	<0.3	<0.3	>10	9.4 ± 5.8	C MS: 12 (2-30) ^##^ NC MS: 12 (2-30)*^##^*
MRI scanner	2T	1.5T	1.5T	1.5T	3T	7T	1.5T
VRS quantification	Manual VRS counting in centrum semiovale.	Semi-automated VRS counting and volume measurements in basal ganglia and centrum semiovale.	Manual VRS counting in brainstem, basal ganglia and centrum semiovale.	Manual VRS counting in brainstem, basal ganglia and centrum semiovale.	Semi-automated VRS counting, area and volume measurements in typical (brainstem, basal ganglia, centrum semiovale) as well as atypical regions.	Manual VRS counting in the brain convexity (handknob), anterior crus, anterior commissure, third ventricle-aqueduct transition including basal ganglia, peduncules.	Manual VRS counting in brainstem, basal ganglia and centrum semiovale.
Association of VRSs with MS	Higher VRS count in MS versus HC.	Significantly higher VRS volume in MS versus HC independent of BPF. No difference in VRS count between MS and HC.	Significantly higher total and centrum semiovale VRS count in MS versus HC.	No significant difference in VRS count between MS and HC.	Significantly higher total and atypical VRS count in MS versus HC.	Significantly higher total, brain convexity and anterior crus VRS count in MS versus HC.	Significantly higher total, basal ganglia and brainstem VRS count, and marginally higher centrum semiovale VRS count in MS versus HC. No difference between C MS and NC MS.
Association of VRSs with clinical or demographic variables	No association with age of onset, physical disability, specific functional system involvement, mono- or polysymptomatic involvement.	No significant association with age, disease duration, physical disability, the time since last relapse or with immunomodulatory treatment.	Significantly higher total and centrum semiovale VRS count in male versus female MS patients.	No significant association with age or gender.	N/A	Significant negative association of VRS with age and disease duration. No association with physical disability.	Marginally significant positive correlation with disease duration. The observed difference in VRS count between MS and HC was not independent from age and sex.
Association of VRSs with brain MRI variables	No significant association with the number of CELs at onset.	Significant association with the occurrence of CELs. No association with BH, BPF and T2L.	N/A	N/A	No significant association with BPF.	Significant negative association with BPF. No association with T2L count.	No significant association with BPF or T2L.

*BH, black hole; BPF, brain parenchymal fraction; C, converter from RRMS to SPMS; CEL, contrast-enhancing lesion; HC, healthy control; IMT, immunomodulatory treatment; MS, multiple sclerosis; NC, non-converter; PPMS, Primary Progressive MS; RRMS, Relapsing-Remitting MS; SPMS, Secondary Progressive MS; VRS, Virchow-Robin Spaces*.

* *Disability was assessed using the Expanded Disability Status Scale in MS patients*.

** *In this study, only the range of the Expanded Disability Status Scale was specified. 20 patients had EDSS < 3.0 and 19 patients had EDSS >4*.

# *In this study, only the age range of MS patients was specified. MS and HC groups were age- and gender-matched*.

## *expressed as median (range)*.

Studies of the association of EPVS with clinical and neuroimaging features of MS showed less consistent results (Table 3). While a few studies showed significant associations of EPVS with age (Kilsdonk et al., 2015), disease duration (Kilsdonk et al., 2015; Cavallari et al., 2018) and sex (Etemadifar et al., 2011), others failed to replicate those findings (Achiron and Faibel, 2002; Wuerfel et al., 2008). Most studies reported no association of EPVS with physical disability (Achiron and Faibel, 2002; Wuerfel et al., 2008; Kilsdonk et al., 2015) or conversion to secondary-progressive MS (Cavallari et al., 2018). A 7T MRI study by Kildonsk et al. found that higher EPVS count was associated with global brain atrophy, while other studies using lower field MRI (i.e., 1.5T or 3T) failed to replicate this finding (Wuerfel et al., 2008; Conforti et al., 2014; Cavallari et al., 2018). Two MRI studies investigated the association of EPVS with clinical relapses and the occurrence of enhancing lesions in MS. A cross-sectional MRI study by Achiron et al. found no association between EPVS of the centrum semiovale and contrast enhancing lesions in MS patients within 3 months of disease onset. Wuerfel et al. showed that EPVS count and volume increased during clinical or radiological relapses, thus supporting the hypothesis that EPVS in MS are associated with neuroinflammatory changes. This study provided for the first time evidence of dynamic perivascular space changes in response to inflammation. Further longitudinal studies are needed to confirm and characterize the pathophysiological correlates of perivascular space changes, and ultimately contribute to clarify the role of EPVS not only in neuroinflammatory but also in neurodegenerative and cerebrovascular diseases.

## Conclusions

The perivascular unit represents a crossroad where neurodegenerative, neuroinflammatory and cerebrovascular mechanisms of brain injury dynamically interact. Those mechanisms gravitate around the glymphatic pathway of clearance, of which the perivascular space represents a crucial component. Although confirmatory studies are warranted to clarify the interaction between those mechanisms of brain injury in humans, seminal work in animal models has pointed to the glymphatic pathway as a potential novel mechanism of neuroprotection with implications for neurodegenerative, neuroinflammatory, and cerebrovascular disorders. The assessment of MRI-visible EPVS dynamics, as well as emerging and more advanced neuroimaging approaches to assess directly glymphatic dynamics may provide surrogate biomarkers to clarify the relevance of the PVU and glymphatic clearance to neurological diseases in humans.

## Author Contributions

FT wrote the review. VC and MM gave their contributions on small vessel disease and AD. EM, MP, VR, and CR helped in the part of neuroimmunology and neuroinflammation. FO, MC, CG, FG, GR, and MS helped in critically reviewing the manuscript.

### Conflict of Interest

The authors declare that the research was conducted in the absence of any commercial or financial relationships that could be construed as a potential conflict of interest.
