# Corrigendum: Perivascular Unit: This Must Be the Place. The Anatomical Crossroad Between the Immune, Vascular and Nervous System

**DOI:** 10.3389/fnana.2020.00051

**Published:** 2020-09-15

**Authors:** Fernanda Troili, Virginia Cipollini, Marco Moci, Emanuele Morena, Miklos Palotai, Virginia Rinaldi, Carmela Romano, Giovanni Ristori, Franco Giubilei, Marco Salvetti, Francesco Orzi, Charles R. G. Guttmann, Michele Cavallari

**Affiliations:** ^1^Department of Human Neuroscience, Sapienza University of Rome, Rome, Italy; ^2^Department of Medicine, Surgery and Dentistry, “Scuola Medica Salernitana”, Neuroscience Section, University of Salerno, Baronissi, Italy; ^3^Department of Neurology and Psychiatry, Sapienza University of Rome, Rome, Italy; ^4^Center for Neurological Imaging, Department of Radiology, Brigham and Women's Hospital, Harvard Medical School, Boston, MA, United States; ^5^Department of Neurosciences, Mental Health and Sensory Organs, Faculty of Medicine and Psychology, Centre for Experimental Neurological Therapies, Sapienza University, Rome, Italy; ^6^Department of Neurosciences Mental Health and Sensory Organs, Faculty of Medicine and Psychology, Sapienza University of Rome, Rome, Italy; ^7^IRCCS Istituto Neurologico Mediterraneo (INM) Neuromed, Pozzilli, Italy

**Keywords:** glymphatic system, perivascular space (PVS), neurodegenaration, neuroinflammation, amyloid, aquaporin (AQP)-4, blood brain barrier (BBB)

In the published article, there was an error in affiliations.

Fernanda Troili and Virginia Cipollini, instead of Department of Neurosciences Mental Health and Sensory Organs, Faculty of Medicine and Psychology, Sapienza University of Rome, Rome, Italy, are affiliated to Department of Human Neuroscience, Sapienza University of Rome, Rome, Italy.

Emanuele Morena, Virginia Rinaldi, Carmela Romano and Francesco Orzi are affiliated to Department of Neurology and Psychiatry, Sapienza University of Rome, Rome, Italy.

Franco Giubile is affiliated to Department of Neurosciences Mental Health and Sensory Organs, Faculty of Medicine and Psychology, Sapienza University of Rome, Rome, Italy.

Giovanni Ristori is affiliated to Department of Neurosciences, Mental Health and Sensory Organs, Faculty of Medicine and Psychology, Centre for Experimental Neurological Therapies, Sapienza University, Rome, Italy.

Marco Salvetti is affiliated to Department of Neurosciences, Mental Health and Sensory Organs, Faculty of Medicine and Psychology, Centre for Experimental Therapies, Sapienza University, Rome, Italy and IRCCS Istituto Neurologico Mediterraneo (INM) Neuromed, Pozzilli, Italy.

The authors apologize for this error and state that this does not change the scientific conclusions of the article in any way. The original article has been updated.

